# Association Between Ankle-Brachial Index and Coronary Artery Calcification Score in Patients Beginning Hemodialysis

**DOI:** 10.7759/cureus.87749

**Published:** 2025-07-11

**Authors:** Kiryu Yoshida, Hirohito Sugawara, Hiroki Mizuyama, Hiroya Shigematsu, Takafumi Fujita, Yoshinori Saito, Masanori Kato, Akiko Takeshima, Masahiro Yamamoto, Hidetoshi Ito

**Affiliations:** 1 Division of Nephrology, Department of Internal Medicine, Showa Medical University Northern Yokohama Hospital, Yokohama, JPN

**Keywords:** abi, ankle-brachial index, cacs, chronic kidney disease, coronary artery calcium score, dialysis, hemodialysis, medial arterial calcification

## Abstract

Introduction

Patients initiating hemodialysis (HD) are at high risk for cardiovascular disease. The ankle-brachial index (ABI) is a simple and widely used tool to detect peripheral artery disease and reflects different patterns of vascular calcification: low ABI indicates intimal arterial calcification, while high ABI may suggest medial arterial calcification (MAC). Coronary artery calcium score (CACS), a marker of coronary atherosclerosis, may reflect both IAC and MAC, though it does not distinguish between them. However, the association between ABI and CACS at HD initiation remains unclear.

Methods

In this single-center cross-sectional study, we included 204 patients who initiated HD and underwent both ABI and coronary CT between November 2013 and December 2023, at the time of dialysis initiation. Patients with ischemic heart disease or prior peritoneal dialysis were excluded to reduce confounding due to coronary interventions or altered baseline parameters. ABI was measured using an automated oscillometric device; the mean of bilateral values was used. ABI was categorized as low (≤0.90), normal (0.91-1.30), or high (>1.30). CACS was compared using the Wilcoxon rank-sum test. Multivariable restricted cubic spline (RCS) models with three knots assessed nonlinear associations between ABI and log-transformed CACS. Logistic regression evaluated the association between the ABI group and high CACS (>400). Models were adjusted for age, sex, diabetes, BMI, smoking status, estimated glomerular filtration rate, serum albumin, CRP, calcium, phosphate, and statin use. Subgroup analyses by sex and diabetes status were also performed.

Results

Median CACS values with IQRs were 930.5 (159.3-2241.1) for the low ABI group, 315.7 (58.5-1046.6) for the normal ABI group, and 54.3 (0.0-413.5) for the high ABI group. Compared to the normal group, low ABI was significantly associated with higher CACS (p = 0.037), while high ABI was associated with lower CACS (p = 0.002). RCS analysis in the unadjusted model showed a curve similar to group-wise comparisons. In the age-adjusted and fully adjusted models, the spline curve in the low ABI range tended to decline. Logistic regression showed a similar trend: low ABI was associated with high CACS in the unadjusted model (OR 2.74, p = 0.029), but the association diminished in the fully adjusted model (OR 1.29, p = 0.651). High ABI was associated with lower odds of high CACS across models, though not statistically significant. Although statistical significance was inconsistent, subgroup analyses stratified by sex and diabetes showed directionally similar trends, and interaction terms in logistic models were not significant.

Conclusions

In patients at the initiation of HD, low ABI (≤0.9) was associated with higher CACS, whereas high ABI (>1.3) was associated with lower CACS. Multivariable analysis indicated that elevated CACS in the low ABI group may be largely influenced by background factors such as age. In contrast, the relatively low CACS in the high ABI group may suggest delayed progression of MAC from peripheral to central arteries, although this remains a hypothesis-generating observation. These findings underscore the potential utility of ABI and CACS as complementary tools for early cardiovascular risk stratification in incident HD patients and may inform future research on vascular calcification dynamics.

## Introduction

Patients undergoing dialysis are at a high risk for cardiovascular disease (CVD) [1], and appropriate assessment of this risk is critical. In our center, ankle-brachial index (ABI) measurement and coronary CT are routinely performed at the initiation of hemodialysis (HD) to evaluate cardiovascular risk. This study examined the association between ABI and coronary artery calcium score (CACS) at HD initiation.

The ABI is a simple and widely adopted tool to detect peripheral artery disease [2]. While low ABI indicates atherosclerotic stenosis due to intimal arterial calcification (IAC), high ABI arises when medial arterial calcification (MAC) stiffens the arterial wall and artificially elevates ankle systolic pressure [3,4]. The relationship between ABI and outcomes is known to follow a U-shaped curve [5-7]. Particularly in patients with chronic kidney disease (CKD), high ABI values are frequently observed [8], and studies in maintenance dialysis support this U-shaped risk relationship [9]. The CACS, as proposed by Agatston et al. [10], is a widely used measure of coronary atherosclerosis and has been shown to predict cardiovascular events [11]. Patients undergoing dialysis tend to have higher CACS compared to the general population [12].

Although several studies have examined the association between ABI and CACS [13-16], low ABI has been consistently associated with high CACS, whereas findings regarding high ABI remain inconclusive. One study in maintenance dialysis patients [17] found that low ABI was associated with higher calcification scores in main arteries (aorta and iliac-femoral axis), while high ABI was associated with calcification in peripheral or distal arteries (pelvic, radial, or digital). However, studies specifically targeting patients at dialysis initiation are few.

This study evaluated the association between three ABI categories (low: ≤0.90, normal: 0.91-1.30, and high: >1.30) and CACS in patients initiating HD to determine whether peripheral arterial calcification at this early stage reflects coronary atherosclerosis burden. Observations from this understudied incident-HD period could help clarify the mechanisms driving IAC versus MAC. Moreover, establishing how the low-cost, simple ABI relates to CACS at HD initiation may provide a cost-effective framework for vascular risk stratification during subsequent dialysis care.

## Materials and methods

Study design and population

We conducted a single-center, cross-sectional study. We screened 273 adult patients who initiated maintenance HD at our center and underwent coronary CT within 30 days before or after HD initiation between November 2013, when CACS measurement first became available at our institution, and December 2023. Patients with a history of ischemic heart disease were excluded because they may have coronary stents, and patients who transitioned from peritoneal dialysis were excluded because their baseline laboratory values and prescriptions may differ. Of these, 204 patients who also had ABI measurements at initiation were included. The study was approved by the institutional ethics committee (approval 2025-0012), and informed consent was obtained through an opt-out process.

Data collection

Patient demographics and baseline clinical data were obtained from electronic medical records. Laboratory values were taken from the day of or immediately before the first dialysis session. Medication use was assessed based on prescriptions immediately prior to dialysis initiation. ABI was measured by trained technicians using a validated device (BP-203RPE III; Omron, Kyoto, Japan). Blood pressure was measured at the upper arm and ankle levels using the oscillometric method in a supine position. ABI was calculated by dividing each ankle systolic pressure by the higher of the two brachial pressures. In patients with a vascular access, the contralateral arm was used for brachial pressure measurement. All ABI measurements were double-checked by a second trained technician, and any questionable values were remeasured. The mean of left and right ABI was used for analysis [17]. CACS was assessed using a 128-slice, non-contrast multidetector-row CT scanner (SOMATOM Definition Flash; Siemens Healthcare, Forchheim, Germany).

Statistical analysis

ABI was categorized into three groups: low (≤0.90), normal (0.91-1.30), and high (>1.30) [2,17]. We performed pairwise comparisons of background characteristics and baseline laboratory and medication data between the normal ABI group and each of the low and high ABI groups. Continuous variables were presented as mean ± SD or median (IQR) and compared using Welch’s t-tests or Wilcoxon rank-sum tests, based on normality assessed by the Shapiro-Wilk test. No outlier treatment was applied. Categorical variables were summarized as frequencies and compared using chi-squared tests or Fisher’s exact tests.

Because CACS followed a right-skewed distribution including zero, group differences in CACS were assessed using Wilcoxon tests with the normal ABI group as reference. The effect size (r) was calculated as the Z value divided by the square root of the total number of observations in the two groups. For regression analysis, CACS was log-transformed as log(CACS + 1). Restricted cubic spline (RCS) regression with three knots (10th, 50th, and 90th percentiles) was used to model the nonlinear relationship between ABI and log(CACS + 1). Nonlinearity was statistically tested by comparing the RCS model with the linear model using a likelihood ratio test. Four models were fitted: unadjusted, age-adjusted, estimated glomerular filtration rate (eGFR)-adjusted (using eGFR at dialysis initiation), and fully adjusted. The fully adjusted model included age, sex, diabetes, BMI, smoking history, eGFR, albumin, CRP, corrected calcium, phosphate, and statin use; only smoking history had missing data (2.5%), which were handled by complete-case analysis. Additionally, logistic regression was performed to evaluate the association between ABI group and high CACS (defined as above 400) using the same four models. The discriminative performance of each logistic regression model was evaluated using the area under the receiver operating characteristic curve. Subgroup analyses by sex and diabetes status were conducted to account for sex-related differences in ABI and the influence of diabetes on MAC.

All analyses were performed using R version 4.3.2, with a two-sided significance level of 0.05.

## Results

**Figure 1:**
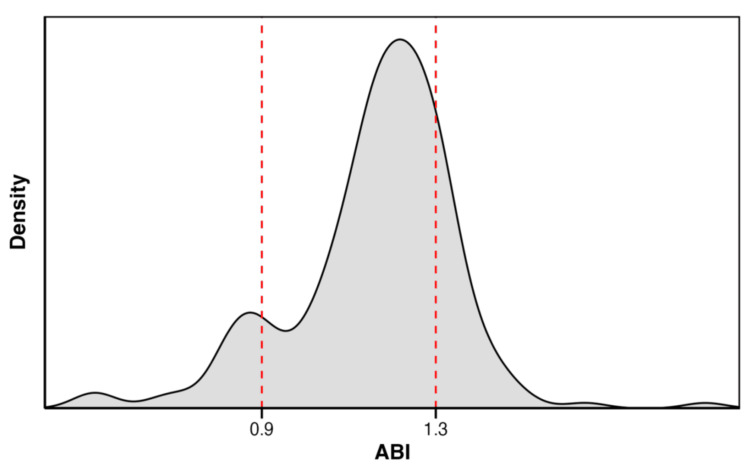
Distribution of ABI among the study population Dashed red lines at 0.90 and 1.30 indicate the thresholds used to define low, normal, and high ABI categories. ABI, ankle-brachial index

Of 273 patients screened, 204 met the inclusion criteria. The distribution of ABI values is shown in Figure 1. The baseline characteristics are summarized in Table 1. The cohort included 30.4% women and 59.8% diabetic patients; mean eGFR at initiation was 5.47 mL/min/1.73 m². Compared with the normal ABI group, patients in the low ABI group were older, had lower BMI, more diabetes and cerebrovascular disease, and higher CRP levels. Patients in the high ABI group were younger than the normal group and also exhibited elevated CRP levels. Serum phosphate and calcium levels did not differ significantly between the low and high ABI groups and the normal ABI group.

**Table 1:** Patient characteristics by ABI groups Data include patient demographics, comorbidities, laboratory values on the day of or immediately before the first dialysis session, and medications prescribed immediately prior to dialysis initiation. P values were calculated using the Welch’s t-test or Wilcoxon rank-sum test for continuous variables and chi-squared test or Fisher’s exact test for categorical variables, as appropriate. ABI, ankle-brachial index; ADPKD, autosomal dominant polycystic kidney disease; Alb, albumin; cCa, corrected calcium; CKD, chronic kidney disease; eGFR, estimated glomerular filtration rate; Hb, hemoglobin; iPTH, intact parathyroid hormone; K, potassium; P, phosphate; RAAS, renin angiotensin aldosterone system; UA, uric acid

Variable	Overall	Low ABI group (≤0.90)	Normal ABI group (0.91-1.30)	High ABI group (>1.30)	p-value (low vs. normal)	p-value (high vs. normal)
n	204	25	142	37		
ABI, median (IQR)	1.19 (1.08-1.28)	0.83 (0.79-0.88)	1.18 (1.11-1.23)	1.35 (1.32-1.41)	<0.001	<0.001
Age, years, median (IQR)	72.50 (61.00-80.00)	78.00 (72.00-82.00)	73.00 (64.00-79.00)	61.00 (51.00-73.00)	0.007	0.003
Sex (male/female) (%)	142/62 (69.6/30.4)	11/14 (44.0/56.0)	100/42 (70.4/29.6)	31/6 (83.8/16.2)	0.02	0.144
BMI, kg/m², median (IQR)	21.95 (19.84-24.58)	19.85 (18.32-22.58)	21.89 (19.86-24.66)	22.87 (20.84-25.08)	0.013	0.142
Smoking history (%)	124 (62.3)	17 (70.8)	81 (57.9)	26 (74.3)	0.267	0.084
Diabetes mellitus (%)	122 (59.8)	20 (80.0)	76 (53.5)	26 (70.3)	0.016	0.093
History of cerebrovascular disease (%)	34 (16.7)	10 (40.0)	18 (12.7)	6 (16.2)	0.002	0.591
Cause of CKD (%)					0.46	0.375
Diabetic kidney disease	103 (50.5)	15 (60.0)	65 (45.8)	23 (62.2)		
Nephrosclerosis	60 (29.4)	9 (36.0)	43 (30.3)	8 (21.6)		
Glomerulonephritis	15 (7.4)	0 (0.0)	13 (9.2)	2 (5.4)		
ADPKD	2 (1.0)	0 (0.0)	1 (0.7)	1 (2.7)		
Other	19 (9.3)	1 (4.0)	16 (11.3)	2 (5.4)		
Unknown	5 (2.5)	0 (0.0)	4 (2.8)	1 (2.7)		
Phosphate binder (%)	63 (30.9)	7 (28.0)	45 (31.7)	11 (29.7)	0.817	1
Vitamin D receptor activator (%)	58 (28.4)	8 (32.0)	43 (30.3)	7 (18.9)	1	0.218
RAAS inhibitors (%)	69 (33.8)	13 (52.0)	45 (31.7)	11 (29.7)	0.067	1
Statin (%)	75 (36.8)	11 (44.0)	49 (34.5)	15 (40.5)	0.374	0.564
Antiplatelet agent (%)	64 (31.4)	15 (60.0)	39 (27.5)	10 (27.0)	0.002	1
eGFR, mL/min/1.73 m², median (IQR)	5.30 (4.10-6.40)	5.90 (4.30-6.90)	5.20 (4.00-6.27)	5.30 (4.30-6.40)	0.147	0.977
Hb, g/dL, median (IQR)	9.20 (8.30-10.10)	9.80 (8.90-10.10)	9.25 (8.40-10.28)	9.00 (8.00-9.80)	0.252	0.08
Alb, g/dL, median (IQR)	3.40 (3.00-3.80)	3.10 (2.90-3.70)	3.40 (3.00-3.80)	3.40 (3.00-3.70)	0.288	0.756
UA, mg/dL, median (IQR)	6.80 (5.70-8.10)	7.00 (5.90-7.70)	6.70 (5.70-8.10)	6.80 (6.10-8.40)	0.709	0.456
K, mEq/L, median (IQR)	4.60 (4.10-5.23)	4.60 (4.10-5.40)	4.60 (4.10-5.20)	4.50 (4.00-5.20)	0.861	0.853
P, mg/dL, median (IQR)	6.00 (5.18-7.30)	5.50 (4.80-7.00)	6.00 (5.03-7.20)	6.00 (5.30-7.80)	0.442	0.217
cCa, mg/dL, median (IQR)	8.40 (7.80-8.90)	8.60 (7.90-9.10)	8.40 (7.80-8.88)	8.40 (7.50-8.80)	0.132	0.984
iPTH, pg/mL, median (IQR)	299.00 (184.00-442.00)	267.00 (151.00-338.00)	305.00 (200.00-439.00)	315.00 (230.00-469.00)	0.146	0.616
CRP, mg/dL, median (IQR)	0.18 (0.07-0.97)	0.52 (0.13-2.63)	0.15 (0.05-0.72)	0.32 (0.13-1.26)	0.026	0.034
Bicarbonate, mEq/L, mean (SD)	19.24 (3.94)	20.52 (3.57)	19.08 (3.55)	18.98 (5.29)	0.071	0.921
Urinary protein, g/gCr, median (IQR)	3.17 (1.87-5.72)	3.13 (1.64-5.28)	3.16 (1.90-5.14)	4.23 (2.06-6.92)	0.941	0.215

CACS comparison across ABI groups

**Figure 2:**
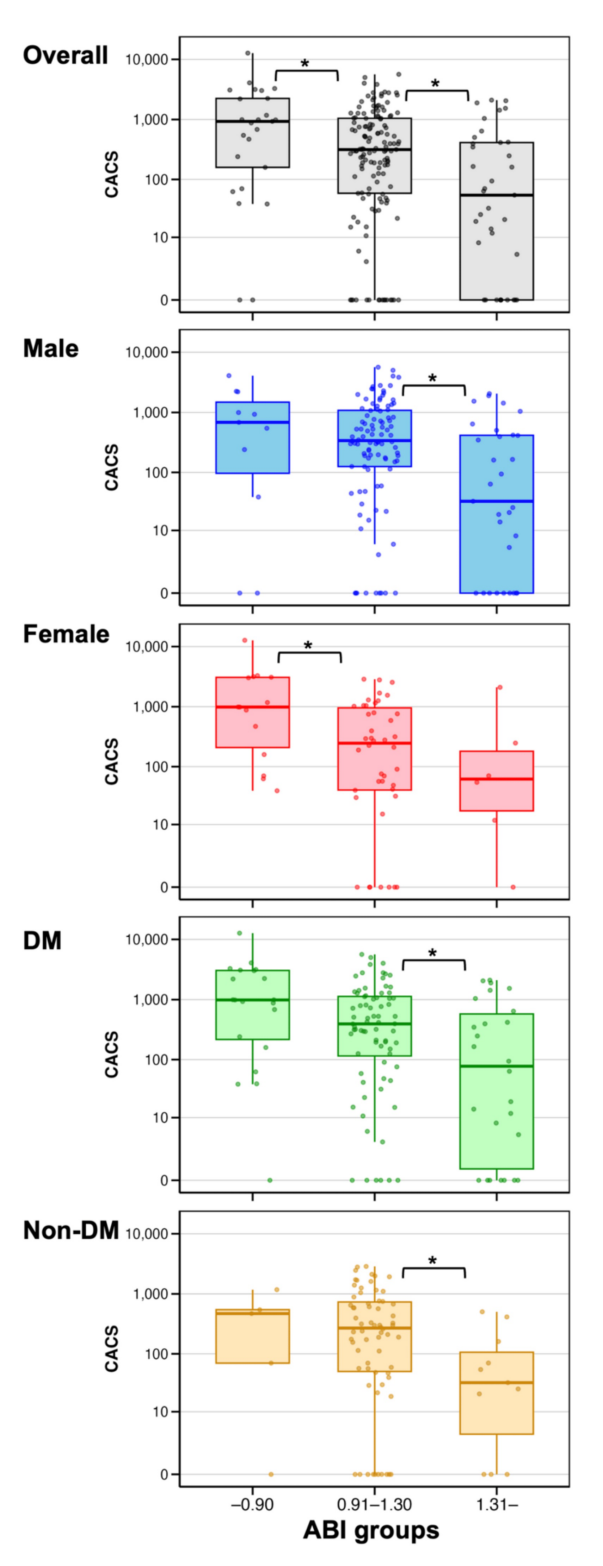
Distribution of CACS by ABI groups Box plots illustrate the distribution of CACS across ABI groups (≤0.90, 0.91-1.30, and >1.30) in the overall cohort and subgroups. Boxes indicate the IQR, the horizontal line within each box represents the median, and whiskers extend to 1.5 times the IQR. The Y-axis is plotted on a log(CACS + 1) scale. Asterisks (*) indicate significant differences (p < 0.05) versus the normal ABI group based on the Wilcoxon rank-sum test. ABI, ankle-brachial index; CACS, coronary artery calcium score; DM, diabetes mellitus

Median CACS values were 930.54 (low ABI), 315.65 (normal ABI), and 54.32 (high ABI) (Figure 2, Table 2). Compared to the normal ABI group, CACS was significantly higher in the low ABI group (p = 0.037) and lower in the high ABI group (p = 0.002). These trends were generally consistent across sex and diabetes subgroups. Among men, high ABI was significantly associated with a lower CACS compared to a normal ABI (p = 0.002), and among women, low ABI was associated with a higher CACS (p = 0.015). In patients with diabetes, the pattern resembled the overall findings, though the difference for low ABI was not statistically significant. In nondiabetic patients, the association between high ABI and lower CACS remained, but the difference between low and normal ABI was not significant.

**Table 2:** CACS comparison across ABI groups CACS were compared between the low and normal ABI group and between the high and normal ABI groups using the Wilcoxon rank-sum test. The effect size (r) was calculated as the Z value divided by the square root of the total number of observations in the two groups. ABI, ankle-brachial index; CACS, coronary artery calcium score; DM, diabetes mellitus

Subgroup	n	Low ABI group (≤0.90)		Normal ABI group (0.91-1.30)		High ABI group (>1.30)		Low vs. normal	High vs. normal
		n (%)	CACS, median (IQR)	n (%)	CACS, median (IQR)	n (%)	CACS, median (IQR)		
Overall	204	25 (12.3)	930.54 (159.30-2241.06)	142 (69.6)	315.65 (58.52-1046.62)	37 (18.1)	54.32 (0.00-413.46)	W = 1310.5, p = 0.037, r = 0.16	W = 3488, p = 0.002, r = 0.23
Male	142	11 (7.7)	683.59 (139.61-1597.80)	100 (70.4)	340.18 (125.36-1082.28)	31 (21.8)	32.50 (0.00-416.25)	W = 484, p = 0.518, r = 0.06	W = 2120.5, p = 0.002, r = 0.27
Female	62	14 (22.6)	987.86 (237.10-3076.36)	42 (67.7)	247.81 (40.21-960.98)	6 (9.7)	62.18 (22.51-203.57)	W = 164.5, p = 0.015, r = 0.33	W = 152.5, p = 0.417, r = 0.12
DM	122	20 (16.4)	987.86 (220.41-3046.23)	76 (62.3)	394.75 (116.13-1129.22)	26 (21.3)	78.81 (1.18-586.44)	W = 548, p = 0.056, r = 0.20	W = 1288, p = 0.021, r = 0.23
Non-DM	82	5 (6.1)	470.51 (69.73-545.53)	66 (80.5)	269.62 (50.48-734.75)	11 (13.4)	32.50 (10.38-115.44)	W = 165.5, p = 1.000, r < 0.01	W = 529, p = 0.016, r = 0.28

RCS analysis

**Figure 3:**
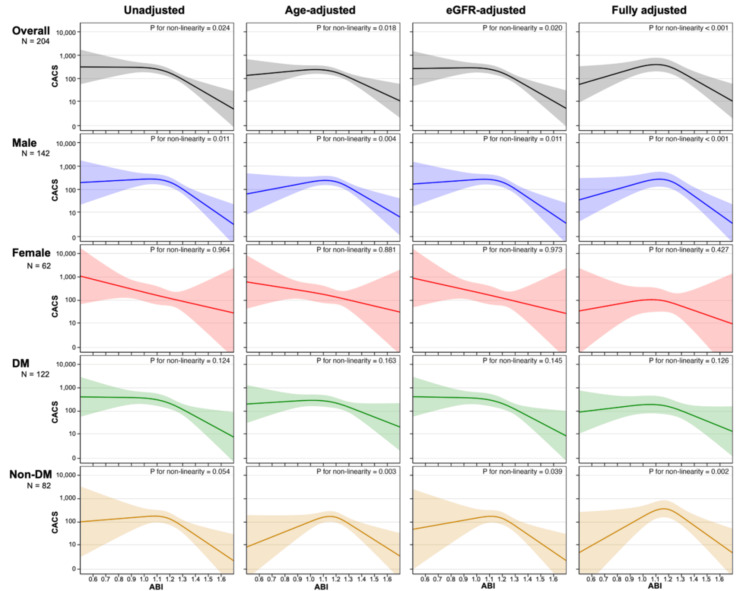
RCS model showing the association between ABI and CACS RCS models with three knots (at the 10th, 50th, and 90th percentiles) were used to examine the association between ABI and log-transformed CACS (log(CACS + 1)). Nonlinearity was statistically tested by comparing the RCS model with the linear model using a likelihood ratio test. Four models were fitted: unadjusted; adjusted for age; adjusted for eGFR at dialysis initiation; and fully adjusted for age, eGFR, sex, DM, BMI, smoking history, albumin, CRP, corrected calcium, phosphate, and statin use. Curves and 95% CIs are shown for the overall cohort and subgroups. ABI, ankle-brachial index; CACS, coronary artery calcium score; DM, diabetes mellitus; eGFR, estimated glomerular filtration rate; RCS, restricted cubic spline

The RCS analysis results are shown in Figure 3, based on four models: unadjusted, age-adjusted, eGFR-adjusted, and fully adjusted (including age, eGFR at dialysis initiation, sex, diabetes, BMI, smoking history, albumin, CRP, corrected calcium, phosphate, and statin use). In the unadjusted model, a tendency toward an inverse pattern between ABI and CACS was observed, which resembled the findings from the group-based comparisons. In the overall population, as well as in male, diabetic, and non-diabetic subgroups, the relationship appeared relatively flat in the lower ABI range but showed a steeper decline beginning around an ABI of 1.1-1.2. Among females, the relationship appeared more linear and consistently inverse. In the age-adjusted and fully adjusted models, the spline curve showed a downward trend in the low ABI range compared to the unadjusted model, whereas the eGFR-adjusted model showed a similar shape to the unadjusted model. Since log(CACS + 1) was used as the dependent variable, the effect estimates should be interpreted on a log scale, reflecting relative changes in CACS. Additionally, as nonlinearity was not statistically significant in some models, these curves should be interpreted with caution.

Logistic regression analysis

Logistic regression (Table 3) evaluated the odds of having a high CACS, defined as above 400. Low ABI was associated with higher odds in the unadjusted model (OR 2.74, p = 0.029). This association lost statistical significance after adjusting for age (OR 2.18, p = 0.098) and also in the fully adjusted model (OR 1.29, p = 0.651), whereas it remained significant after adjusting for eGFR alone (OR 2.53, p = 0.046). High ABI was associated with lower odds in the unadjusted model (OR 0.48, p = 0.070), and although not statistically significant in any model, the inverse association was consistently observed across all levels of adjustment. Subgroup analyses showed that the association between low ABI and higher odds of high CACS was particularly pronounced in females (unadjusted OR 4.50, p = 0.026), consistent with the findings from the group-wise comparisons and RCS analysis. However, formal interaction tests revealed that the interaction terms between ABI and sex, as well as between ABI and diabetes status, were not statistically significant in any of the models (p > 0.05).

**Table 3:** Logistic regression analysis of the association between ABI groups and high CACS High CACS was defined as greater than 400. ORs and 95% CIs were calculated using logistic regression. The normal ABI group (0.91-1.30) was used as the reference. Four models were applied: unadjusted; adjusted for age; adjusted for eGFR at dialysis initiation; and fully adjusted for age, eGFR, sex, DM, BMI, smoking history, albumin, CRP, corrected calcium, phosphate, and statin use. An asterisk (*) indicates models with AUC < 0.6, suggesting poor discriminatory performance. ABI, ankle-brachial index; AUC, area under the curve; CACS, coronary artery calcium score; DM, diabetes mellitus; eGFR, estimated glomerular filtration rate

Subgroup	Unadjusted			Age-adjusted			eGFR-adjusted			Fully adjusted		
	Low ABI group (≤0.90)	Normal ABI group (0.91-1.30)	High ABI group (>1.30)	Low ABI group (≤0.90)	Normal ABI group (0.91-1.30)	High ABI group (>1.30)	Low ABI group (≤0.90)	Normal ABI group (0.91-1.30)	High ABI group (>1.30)	Low ABI group (≤0.90)	Normal ABI group (0.91-1.30)	High ABI group (>1.30)
Overall	2.74 (1.11-6.77) p = 0.029	1.00 (reference)	0.48 (0.22-1.06) p = 0.070	2.18 (0.87-5.47) p = 0.098	1.00 (reference)	0.61 (0.26-1.39) p = 0.237	2.53 (1.01-6.31) p = 0.046	1.00 (reference)	0.49 (0.22-1.09) p = 0.081	1.29 (0.43-3.88) p = 0.651	1.00 (reference)	0.41 (0.16-1.06) p = 0.067
Male	1.97 (0.54-7.17) p = 0.302^*^	1.00 (reference)	0.46 (0.19-1.10) p = 0.081^*^	1.31 (0.35-4.95) p = 0.693	1.00 (reference)	0.61 (0.24-1.51) p = 0.284	1.80 (0.49-6.64) p = 0.378^*^	1.00 (reference)	0.48 (0.20-1.15) p = 0.100^*^	0.85 (0.18-4.05) p = 0.840	1.00 (reference)	0.42 (0.15-1.17) p = 0.097
Female	4.50 (1.20-16.85) p = 0.026	1.00 (reference)	0.36 (0.04-3.37) p = 0.371	4.35 (1.14-16.55) p = 0.031	1.00 (reference)	0.39 (0.04-3.79) p = 0.416	4.07 (1.06-15.54) p = 0.040	1.00 (reference)	0.35 (0.04-3.36) p = 0.365	1.85 (0.28-12.34) p = 0.524	1.00 (reference)	0.20 (0.01-4.41) p = 0.307
DM	2.46 (0.85-7.08) p = 0.095	1.00 (reference)	0.47 (0.18-1.21) p = 0.116	2.00 (0.67-5.95) p = 0.213	1.00 (reference)	0.53 (0.20-1.39) p = 0.198	2.48 (0.86-7.17) p = 0.094	1.00 (reference)	0.48 (0.18-1.24) p = 0.128	1.83 (0.50-6.68) p = 0.358	1.00 (reference)	0.45 (0.15-1.37) p = 0.158
Non-DM	2.46 (0.38-15.76) p = 0.342^*^	1.00 (reference)	0.36 (0.07-1.82) p = 0.219^*^	1.51 (0.23-10.10) p = 0.671	1.00 (reference)	0.61 (0.10-3.57) p = 0.582	1.68 (0.22-12.61) p = 0.614^*^	1.00 (reference)	0.39 (0.08-1.99) p = 0.258^*^	0.41 (0.03-6.64) p = 0.533	1.00 (reference)	0.51 (0.07-3.55) p = 0.498

## Discussion

In this study of patients initiating HD, a low ABI (≤ 0.9) was associated with a higher CACS, whereas a high ABI (> 1.3) was associated with a lower CACS. In the RCS analyses and logistic regression, adjustment for age and in the fully adjusted models attenuated the association at low ABI levels, with the relationship shifting toward a downward trend in CACS.

CACS is widely used to assess coronary artery calcification, but arterial calcification can result from two distinct mechanisms: IAC and MAC [4,18]. IAC causes arterial lumen narrowing due to lipid deposition and inflammation, resulting in a low ABI [4,19]. Our findings showed that a low ABI (≤0.9) was associated with high CACS; however, patients in the low ABI group were older. Age is a known risk factor for IAC [19]. Accordingly, the association was attenuated in age-adjusted models, suggesting a substantial influence of confounding by age.

MAC, in contrast, leads to arterial stiffening without lumen narrowing and is often associated with a high ABI [3]. CKD is a known risk factor for MAC [4], yet in our study of patients at the initiation of dialysis, high ABI was paradoxically associated with a low CACS. This result remained even after adjustment, suggesting it may not be due to confounding. It has been reported that the risk of MAC increases with dialysis duration [20], and MAC may progress from peripheral arteries to central arteries such as the coronary arteries [18]. In this study focusing on patients at the time of initiating dialysis, such a time lag may explain the observed findings. A study of maintenance dialysis patients [17] reported that the OR for main artery calcification in those with ABI >1.3 was above 1, though not statistically significant. This discrepancy may reflect a more advanced progression of MAC to central arteries in maintenance dialysis patients compared to those at the time of initiating dialysis.

These interpretations remain speculative and should be regarded as hypotheses rather than definitive conclusions. CACS cannot distinguish between IAC and MAC [21]. Prior research has suggested that distinct calcification patterns on CT may help differentiate IAC from MAC [22]. Additionally, intravascular imaging techniques, including intravascular ultrasound and optical coherence tomography, may offer more precise characterization [23]. Future studies incorporating these approaches are warranted to clarify the specific contributions of IAC and MAC in dialysis patients.

ABI tends to be slightly lower in women due to smaller body size [24,25]. In our study, however, the interaction between ABI and sex in relation to CACS was not statistically significant. In our cohort, the number of low ABI cases was limited among men, and high ABI cases were few among women. This imbalance may explain why statistically significant trends observed in the overall cohort were not consistently reproduced within each sex subgroup. Regarding diabetes status, the prevalence of diabetes was significantly higher in the low ABI group compared to the normal ABI group and also somewhat higher in the high ABI group, although not statistically significant. The elevated CRP levels observed in these groups may be partially attributable to the distribution of diabetes [26]. While diabetes, like CKD, is a known risk factor for MAC [4], it is also thought to contribute to IAC through mechanisms such as chronic inflammation and oxidative stress [27]. However, the interaction between ABI and diabetes status in relation to CACS was not statistically significant, and the results in diabetic patients were broadly consistent with those in the overall population. In the non-diabetic subgroup, the number of low ABI cases was extremely small. Therefore, the interpretation of the downward trend in the spline curve and the lower OR observed in this subgroup requires caution. Further studies with larger sample sizes are warranted to clarify the relationship between ABI and CACS across sex and diabetes subgroups.

This study has several limitations. First, it was a single-center study conducted in Japanese patients without a history of coronary artery disease, which may limit the generalizability of the findings. Although ABI and coronary CT were performed in many patients, they were not conducted in all cases, as some patients were excluded based on clinical judgment or social circumstances. Thus, selection bias due to physician discretion cannot be ruled out. Compared with national registry data on incident dialysis patients in Japan [28], the mean age (72 years) and proportion of female patients (30%) in this study were comparable. However, the high prevalence of diabetic nephropathy and nephrosclerosis, and the low prevalence of other etiologies such as chronic glomerulonephritis, may reflect selective ABI testing and limit generalizability. Second, in some cases, ABI was measured after the creation of vascular access. In such cases, brachial pressure from the contralateral arm was used for calculation. For example, if the arm with better vascular condition was used for shunt creation, the measured ABI might be falsely elevated, potentially failing to reflect the true ABI. Third, we used fixed ABI cutoffs of ≤0.90 and >1.30 for all patients, without accounting for sex-specific thresholds, despite known physiological differences in ABI between men and women. This may have influenced classification accuracy and introduced misclassification bias. Fourth, although we adjusted for multiple clinical variables, residual confounding from unmeasured factors may have influenced the observed associations. Finally, the overall sample size may not have been sufficient, especially within the low and high ABI groups, limiting statistical power. The RCS analyses may have produced statistically unstable curve shapes, warranting cautious interpretation. Additionally, some logistic models, especially in subgroups, showed poor performance and should be interpreted with caution. Larger, multicenter studies are needed to validate these findings in patients initiating HD.

## Conclusions

In this study of patients at the initiation of HD, low ABI (≤0.9) was associated with higher CACS, and high ABI (>1.3) with lower CACS. The elevated CACS observed in the low ABI group may have been partly influenced by background factors such as age, as the association attenuated and lost statistical significance after adjustment. In contrast, the relatively low CACS in the high ABI group may reflect a delayed progression of MAC from peripheral to coronary arteries; however, this remains speculative and hypothesis-generating.

Given that CACS cannot distinguish between intimal and medial calcification, and considering the cross-sectional design, single-center setting, modest sample size, and potential selection bias, the generalizability of our findings is limited. The inverse association observed in the high ABI group, in particular, warrants further investigation, as it may reflect different pathophysiological mechanisms. Further validation is needed before ABI can be applied clinically for coronary risk stratification in this population.
